# Discriminatory Value of Adiponectin to Leptin Ratio for COVID-19 Pneumonia

**DOI:** 10.1155/2022/9908450

**Published:** 2022-04-26

**Authors:** Federica Tonon, Stefano Di Bella, Fabiola Giudici, Verena Zerbato, Ludovica Segat, Raffaella Koncan, Andrea Misin, Barbara Toffoli, Pierlanfranco D'Agaro, Roberto Luzzati, Bruno Fabris, Stella Bernardi

**Affiliations:** ^1^Department of Medical Surgical and Health Sciences, University of Trieste, Trieste 34149, Italy; ^2^SC Malattie Infettive, Ospedale Maggiore, Azienda Sanitaria Universitaria Isontino-Giuliana, Trieste 34125, Italy; ^3^Gustave-Roussy, Bureau Biostatistique et Epidémiologie - 114, Rue Eduard Vaillant, Villejuif 94805, France; ^4^U.C.O. Igiene e Sanità Pubblica, Ospedale Maggiore, Azienda Sanitaria Universitaria Isontino-Giuliana, Trieste 34125, Italy; ^5^SS Endocrinologia, UCO Medicina Clinica, Ospedale di Cattinara, Azienda Sanitaria Universitaria Isontino-Giuliana, Trieste 34149, Italy

## Abstract

**Purpose:**

Obesity is a risk factor for severe coronavirus disease 2019 (COVID-19). Circulating adipokines have been associated with inflammatory burden and amplified or dysregulated immune responses. This study aimed to evaluate the discriminatory ability of adipokines to identify COVID-19 pneumonia and to assess disease severity.

**Methods:**

We conducted an observational case-control study, with a prospective design, and recruited patients with diagnosis of COVID-19 pneumonia (*n* = 48) and healthy controls (*n* = 36), who were matched by age, sex, and BMI. Leptin, adiponectin, IL-6, and TNF-*α* were measured by ELISA.

**Results:**

Patients with COVID-19 pneumonia had higher levels of leptin, lower adiponectin/leptin (Adpn/Lep) ratio, and higher expression of IL-6. Leptin had an acceptable discriminatory accuracy for COVID-19 pneumonia in patients with BMI >30 (AUC 0.74 [0.58, 0.90]) with a cutoff of 7852 pg/mL and it was associated with maximum respiratory support. By contrast, Adpn/Lep had an excellent discriminatory accuracy for COVID-19 pneumonia in patients with BMI <25 (AUC 0.9 [0.74, 1.06]) with a cutoff of 2.23.

**Conclusion:**

Our data indicate that high Adpn/Lep (>2.23) in lean patients is consistent with a state of good health, which decreases in case of inflammatory states, ranging from adipose tissue dysfunction with low-grade inflammation to COVID-19 pneumonia.

## 1. Introduction

The year 2019 will be remembered for the deadly outbreak of coronavirus disease 19 (COVID-19), a pandemic due to the severe acute respiratory syndrome coronavirus 2 (SARS-CoV-2). With the worldwide spread of SARS-CoV-2, obesity has emerged as an important determinant of COVID-19 prognosis [1]. Obesity is an independent risk factor for the development of severe COVID-19, requiring intensive care support [2, 3], and leading to higher death rates in hospitalized patients [4, 5]. We have recently demonstrated that neck circumference predicts mortality in hospitalized COVID-19 patients [6].

The relationship between obesity and worse clinical outcomes in viral infections is not new [7]. Frydrych et al., for instance, suggested that this association could be due to an immune system dysfunction triggered by the chronic low-grade inflammation that characterizes obesity [7, 8]. Adipose tissue is an endocrine organ capable of synthesizing a wide variety of molecules called adipokines. When it enlarges, there is a change in adipocyte size, an increase of free fatty acids and tissue hypoxia. These changes are accompanied by a modification of adipose tissue secretory profile and macrophage infiltration, leading to a moderate but chronic release of acute-phase proteins, proinflammatory adipokines, and adhesion molecules [9–11], which could contribute to the development of cytokine storm and cytokine release syndrome, which are life-threatening systemic inflammatory syndromes that can be triggered by various conditions/agents such as SARS-CoV-2 infection [12].

In addition to the proimmunogenic state of adipose tissue in obesity, adipose tissue cells can be targets of multiple viruses, including SARS-CoV-2, because they express ACE2, which is the main receptor of SARS-CoV-2 [13]. Interestingly, patients with obesity exhibit an overexpression of ACE2 as well as of other alternative SARS-CoV-2 receptors (CD147, DPP4, and NRP1) in their visceral fat, increasing their susceptibility to viral infection [14]. This could lead to prolonged viral shedding in this organ, with extended activation of local immune cells and inflammatory response [13, 14].

Leptin and adiponectin are the two best known adipokines produced by the adipose tissue. In physiological conditions, circulating concentrations of leptin are around 2–8 ng/mL [15], while those of adiponectin are around 5–30 *µ*g/mL [16, 17]. These values may vary, due to sex differences, with leptin and adiponectin levels being higher in women [18], as well as due to the presence of comorbidities [19], or to the methods used for their measurement [20, 21]. Nevertheless, leptin is directly associated with stored body fat [15], while adiponectin is inversely related to it [19, 22]. In addition, leptin has proinflammatory actions [23], while adiponectin has anti-inflammatory properties, such as the inhibition of macrophage activity and their production of TNF-*α* [24]. Consequently, these adipokines appear to be the link between obesity, unhealthy adipose tissue, low-grade inflammation, and cardio-metabolic alterations. In this setting, the adiponectin to leptin ratio (Adpn/Lep) has been recently proposed as a marker of adipose tissue dysfunction [23]. Consistent with this concept, it has been demonstrated that the adipocyte dysfunction induced by SARS-CoV-2 infection was associated with a decrease in Adpn/Lep ratio and an upregulation of factors involved in the innate immune response [25].

Based on this background, in this study, we aimed to evaluate whether leptin, adiponectin, as well as Adpn/Lep could help discriminate the presence of COVID-19 pneumonia as well as assess disease severity.

## 2. Materials and Methods

### 2.1. Study Design

This is an observational case-control study, with a prospective design, aiming at evaluating the role of adipokines in patients with COVID-19 of different severity as compared to healthy controls. Between October 2020 and April 2021, patients with diagnosis of COVID-19 pneumonia (cases), who were admitted to the Institute of Malattie Infettive (ASUGI), were consecutively recruited for this study, while age- and sex-matched healthy volunteers with no history of COVID-19 and/or other intercurrent diseases (controls) were recruited at the Institute of Medicina Clinica (ASUGI).

Diagnosis of COVID-19 pneumonia was based on the following criteria: (i) presence of SARS-CoV-2 nucleic acids as assessed by real-time RT-PCR from nasopharyngeal swabs and (ii) respiratory distress (respiratory rate >30 breaths/min), (iii) mean oxygen saturation ≤93%, and (iv) arterial oxygen pressure/oxygen concentration (PaO_2_/FiO_2_) ≤300 mmHg, as well as (*v*) typical ground-glass pulmonary opacities. Exclusion criteria: age <18 years, refusal to participate in the study, and for controls, history or presence of COVID-19 and/or intercurrent diseases.

All participants gave informed consent to participate in this study. In patients with COVID-19 pneumonia (cases), the following parameters were collected: demographics, medical history, medication, vital signs, body mass index, PaO_2_/FiO_2_, complete white blood cell count, CRP, estimated GFR, fasting glucose, and lipid levels. During hospitalization, we recorded the medication prescribed, the maximum respiratory support given, days of noninvasive mechanical ventilation, time to negativity, and death. The maximum respiratory support given was dived into two groups: venturi mask/high-flow nasal cannula (VM-HFNC) group and continuous positive airway pressure (CPAP)/noninvasive mechanical ventilation (NIMV) group. In control patients, the following parameters were collected: demographics, vital signs, body mass index, complete white blood cell count, estimated GFR, fasting glucose, and lipid levels.

In all patients, a fasting blood sample was collected to measure adipokines the day after hospital admission.

This study was conducted in accordance with the Declaration of Helsinki, and it was approved by our Institutional Review Board and regional Ethics Committee on 27/09/2019 (CEUR-2019-SPER-113). All subjects gave their written informed consent before participation in the study.

### 2.2. Adipokine Measurement

Leptin (DY398-05, R&D Systems), adiponectin (DY1065-05, R&D Systems), IL-6 (DY206-05, R&D Systems), and TNF-*α* (DY210-05, R&D Systems) were measured by ELISA, according to manufacturer's instructions. The intra-assay coefficients of variations were 6.39% for leptin, 6.96% for adiponectin, 5.37% for IL-6, and 3.72% for TNF-*α*. The interassay coefficients of variations were 7.26% for leptin, 7.30% for adiponectin, 11.40% for IL-6, and 8.74% for TNF-*α*.

### 2.3. Statistical Analysis

All statistical analyses were carried out in R system for statistical computing (Ver 5.0; R Development Core Team, 2018). Statistical significance was set at *p* < 0.05.

Shapiro-Wilk test was applied to quantitative (continuous) variables to check for distribution normality. Continuous variables were reported as median with range (min-max) or mean ± standard deviation, depending on their distribution. Categorical variables were reported as absolute frequencies and/or percentages. Continuous variables were compared by Mann–Whitney test or *t*-test, depending on data distribution. Linear associations were evaluated with the Pearson or Spearman coefficient. Two logistic multivariate regression models were performed: the first to evaluate factors associated with COVID-19 pneumonia and the second to identify variables related to maximum respiratory support.

Receiver operating characteristic (ROC) curves were constructed to evaluate the sensitivity and specificity of leptin and Adpn/Lep in predicting COVID-19 pneumonia. The area under the curve (AUC) was calculated, with higher values indicating better discriminatory ability. Sensitivity, specificity, and positive and negative predictive values with 95% confidence interval (CI) were then calculated. Areas under the curve (AUCs) for Adpn/Lep respect to BMI were compared using DeLong's test implemented in the R package pROC.

## 3. Results

### 3.1. Patient General Characteristics

A total of 48 patients with COVID-19 pneumonia and 36 healthy controls were recruited. Groups did not differ in terms of age, sex, BMI, and presence of metabolic syndrome, as assessed based on the International Diabetes Federation criteria [20], as shown in Table 1. Patients with COVID-19 pneumonia were on average 64 years old; they were predominantly men (71%) and overweight, as their mean BMI was 29. On admission, their median CRP was 70 mg/L (4–220). The PaO_2_/FiO_2_ was less than 100 in 35.4% of them, 100 to 200 in 35.4%, 200 to 300 in 29.2%, and greater than 300 only in 4.2% of them. All these patients required oxygen support during hospitalization; the maximum respiratory support given was recorded and a total of 32/48 patients (66.67%) received VM/HFNC (Venturi mask/high-flow nasal cannula), while a total of 16/48 patients (33.33%) received NIMV (continuous positive airway pressure/noninvasive mechanical ventilation) in the sub-ICU/ICU for 8.5 days. During hospitalization, patients received preventive (24/48; 50%) or therapeutic antithrombotic therapy (24/48; 50%), corticosteroids (47/48; 97.9%), and/or remdesivir (9/48; 18.75%). The median duration of hospital stay was 10 days (3–49).

### 3.2. Patients with COVID-19 Pneumonia Exhibit Higher Levels of Leptin, Lower Levels of Adpn/Lep, and Higher Levels of IL-6

#### 3.2.1. Leptin

**Figure 1:**
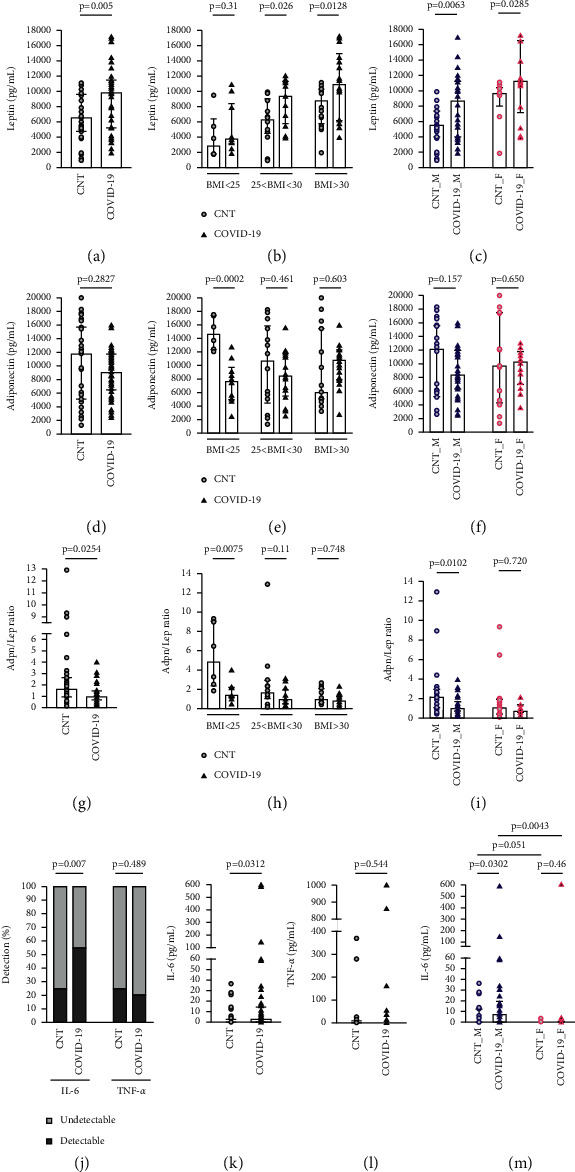
Leptin, adiponectin, Adpn/Lep levels, IL-6, and TNF-*α* in patients with COVID-19 pneumonia (COVID-19) and healthy controls (CNT). (a) Leptin, Mann–Whitney test. (b-c) Leptin after BMI and sex stratification, Mann–Whitney test or unpaired t-test. (d) Adiponectin, *t*-test. (e-f) Adiponectin after BMI and sex stratification, *t*-test. (g) Adpn/Lep, Mann–Whitney test. (h-i) Adpn/Lep after BMI and sex stratification, Mann–Whitney test. (j) Percentage of patients with detectable levels of IL-6 and TNF-*α*, chi-square test. (k) IL-6, Mann–Whitney test. (l) TNF-*g*, Mann–Whitney test. (m) IL-6 after sex stratification, Mann–Whitney test.

In all the patients recruited, we measured leptin, adiponectin, IL-6, and TNF-*α*. Patients with COVID-19 pneumonia exhibited a significant increase of leptin, whose median was 9855 pg/mL (1869–17193), as compared to 6593 pg/mL (915–11114) in healthy controls (Figure 1). Leptin was not associated with age (rho 0.003; *p*=0.97) or sex, but it was associated with BMI (rho 0.54; *p* < 0.0001). When we stratified our population based on their BMI, only patients with a BMI >25 exhibited a significant increase of leptin in case of COVID-19 pneumonia, as shown in Figure 1. In particular, in the subgroup with BMI <25, leptin was 2841 pg/mL (1750–9465) in the control group and 3790 pg/mL (1869–10900) in the COVID-19 group (*p*=0.31). In the subgroup with BMI between 25 and 30, leptin was 6278 pg/mL (915–9857) in the control group and 9336.94 pg/mL (3864–12024) in the COVID-19 group (*p*=0.03). In the subgroup of patients with BMI >30, leptin was 8764.32 pg/mL (1932–11115) in the control group and 10900 pg/mL (3905–17193) in the COVID-19 group (*p*=0.01).

#### 3.2.2. Adpn/Lep

**Figure 2:**
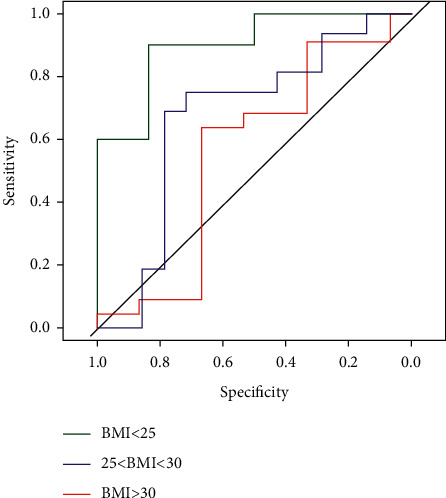
Discriminatory value of Adpn/Lep for COVID-19 pneumonia. Adpn/Lep receiver operator characteristic curves illustrating the ability of Adpn/Lep to identify COVID-19 pneumonia after BMI stratification.

Adiponectin was not associated with sex, age, and BMI. It did not change between healthy subjects and patients with COVID-19 pneumonia, apart from the subgroup of patients with BMI <25 (Figure 1). We also looked at the ratio adiponectin/leptin (Adpn/Lep) [26]. Adpn/Lep did not correlate with age, but it was associated with sex, being higher in male subjects (*p*=0.03) and inversely related to BMI (rho = −0.46; *p* < 0.0001). In our study, Adpn/Lep was 1.65 (0.13–12.9) in the control group and it decreased significantly to 0.98 (0.26–3.98) in patients with COVID-19 pneumonia (*p*=0.025). Nevertheless, when we stratified our population based on BMI, it was only in the subgroup of patients with BMI <25 that we observed a significant change of this ratio in case of COVID-19 pneumonia (Figure 2).

#### 3.2.3. IL-6 and TNF-*α*

The levels of IL-6 and TNF-*α* were not always detectable, so we looked at the percentage of patients with detectable levels (vs those with undetectable levels). The percentage of patients with COVID-19 pneumonia who expressed detectable levels of IL-6 (but not TNF-*α*) was significantly higher than that of controls (*p*=0.0055), as shown in Figure 1. Patients with detectable levels of IL-6 were older (*p*=0.04), and they were more often males (47% of males expressed it as compared to 29% of females; *p*=0.12), as previously reported [27].

### 3.3. Discriminatory Cutoffs of Leptin and Adpn/Lep for COVID-19 Pneumonia

#### 3.3.1. Discriminatory Accuracy of Leptin

Logistic multivariate regression models suggested that leptin and Adpn/Lep were independently associated with the presence of COVID-19 pneumonia (Table 2). ROC curve analysis showed that leptin had an acceptable discriminatory accuracy for COVID-19 pneumonia, being the AUC = 0.68 (0.57, 0.79). The optimal cutoff value of leptin discriminating the presence of COVID-19 pneumonia was 7852 pg/mL, allowing to identify correctly 64.29% of the patients, with a sensitivity of 66.67% and a specificity of 61.11%. After BMI stratification, we found that the AUC increased. The AUC was 0.66 (0.39, 0.99) in patients with BMI <25, it was 0.70 (0.51, 0.89) in patients with BMI ≥25 and <30, and it was 0.74 (0.58, 0.90) in patients with BMI ≥30.

#### 3.3.2. Discriminatory Accuracy of Adpn/Lep

ROC curve analysis showed that Adpn/Lep had a low accuracy to predict COVID-19 pneumonia, being the AUC = 0.64 (0.51, 0.77). Nevertheless, when we looked at the AUCs after BMI stratification, we found that they differed significantly (*p*=0.03; DeLong test). As opposed to leptin, here we found that the best AUC was seen in patients with BMI <25. In particular, in patients with BMI <25, the AUC was 0.9 (0.74, 1.06), while it was 0.67 (0.45, 0.88) in patients with BMI ≥25 and <30, and 0.55 (0.34, 0.77) in patients with BMI >30. In lean subjects (BMI <25), the optimal cutoff value discriminating the presence of COVID-19 pneumonia was 2.23, allowing to identify correctly 88% of the patients with a sensitivity of 0.90 (0.55–0.99) and a specificity of 0.83 (0.36–0.99). NPV = 0.83 (0.41–0.99) and a PPV = 0.90 (0.50–0.99).

### 3.4. Associations between Adipokines and Outcomes of COVID-19 Pneumonia

**Figure 3:**
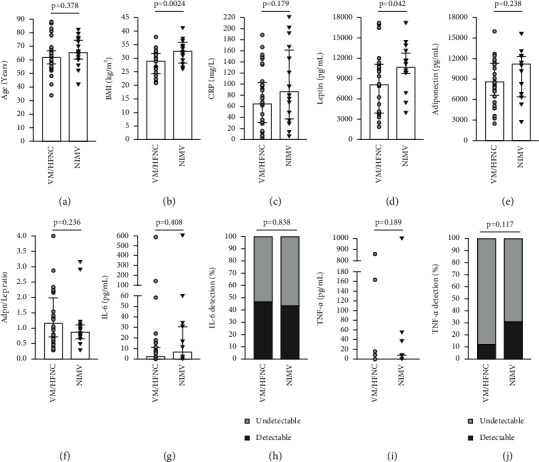
Factors associated with maximum respiratory support given. Mann–Whitney test or *t*-test (a–g), (i), and chi-square test (h, j).

Consistent with our previous data, also in the subgroup of patients with COVID-19 pneumonia, leptin was directly associated to BMI (rho = 0.592; *p* < 0.0001), and Adpn/Lep was inversely related to it (rho = −0.354; *p*=0.014). When we looked at the immune response of patients with COVID-19 pneumonia, the number of leukocytes and lymphocytes was inversely related to IL-6 and TNF-*α* (Table 3). When we looked at the maximum respiratory support given, which we divided into VM/HFNC and NIMV, only BMI and leptin were related to it, being significantly higher in the NIMV group (Figure 3). BMI was also associated with the days of NIMV, while age and BMI were associated with the days of hospital stay. Logistic multivariate regression models suggested that age and BMI, but not leptin, were independently associated with the maximum respiratory support given in patients with COVID-19 pneumonia (Table 4). Consequently, we did not look for the discriminatory cutoffs of leptin and Adpn/Lep for outcomes of COVID-19 pneumonia.

## 4. Discussion

This study shows for the first time that Adpn/Lep had an excellent discriminatory accuracy for the presence of COVID-19 pneumonia in lean people. Adpn/Lep is a ratio that was initially proposed as a marker of adipose tissue dysfunction, increased cardio-metabolic risk [26, 28], and low-grade inflammation [29]. Obesity [15, 22], insulin resistance [19], and inflammation [30–32] are associated with a decrease of adiponectin and an increase of circulating leptin. Consistent with this, Adpn/Lep decreases with the increasing number of metabolic risk factors [28] and it is negatively correlated with CRP levels and systemic inflammation [29]. For these reasons, it has been suggested that Adpn/Lep might discriminate between the patients that are “metabolically healthy” and those that are “metabolically unhealthy” [28].

Frühbeck et al. have recently proposed that Adpn/Lep should have a value >1 in normal conditions, corresponding to a low cardio-metabolic risk, while values between >0.5 and <1 should correspond to a moderate risk, and values <0.5 should correspond to a high cardio-metabolic risk [33]. Interestingly, in this work by Frühbeck et al., lean subjects (mean BMI of 22), with mean arterial blood pressure of 123/71 mmHg, mean glycemia of 91 mg/dL, and lipids levels within reference targets, had a mean value of Adpn/Lep of 2.83 ± 2.16 [33]. In line with these findings, in our study, 2.23 was the optimal cutoff value discriminating between lean patients that were in good health and those with COVID-19 pneumonia, allowing to identify correctly 88% of the patients with a sensitivity of 90% and a specificity of 83%. In other words, a high value of Adpn/Lep is consistent with a state of good health in lean patients and it could rule out any low-grade or acute systemic inflammation, including the state triggered by SARS-CoV-2 infection.

Our study shows that the Adpn/Lep could be used not only to identify patients with adipose tissue dysfunction and high cardio-metabolic risk but also to rule out the presence of acute inflammatory diseases, such as COVID-19 pneumonia. This is consistent with other authors' recent findings [34]. The issue that remains to be solved is the heterogeneity of methods to measure Adpn/Lep, mostly due to the variety of methods available to measure adiponectin [20, 21]. Adiponectin can be measured with different techniques, such as radioimmunoassays, enzyme-linked immunosorbent assays, chemiluminescent immunoassays, and immunoturbidimetric methods, in both manual or fully automated ways [21]. Consequently, a standardization of adiponectin assays, as well as a clear definition of normal and pathological reference ranges is still lacking [20].

It can be speculated that the decrease of Adpn/Lep, which is seen in obese patients and patients with COVID-19 pneumonia, might contribute to development of severe form of disease, due to the anti-inflammatory actions of adiponectin and the proinflammatory effects of leptin. Several studies demonstrate that obesity is a risk factor for developing a severe form of COVID-19 pneumonia [1], requiring intensive care unit admission, intubation, and mechanical ventilation [3, 35–37]. Last year, we demonstrated that also other anthropometric indexes, such as neck circumference, might predict the need of mechanical ventilation support [38]. Consistent with the literature, in this study, we found that BMI, as well as age, was independently associated with the need of noninvasive mechanical ventilation (NIMV), the days of NIMV, and the days of hospitalization. Nevertheless, we did not find any significant association between the levels of circulating adipokines and these outcomes, possibly due to the small number of patients.

The limitations of this study include the small number of patients recruited, as well as the measurement of the adipokines with manual enzyme-linked immunosorbent assays. On the other hand, it has to be taken into account that even in our small cohort, Adpn/Lep had an excellent discriminatory accuracy in identifying COVID-19 pneumonia in lean subjects.

In conclusion, our data indicate that patients with COVID-19 pneumonia had higher levels of leptin and lower Adpn/Lep. Leptin had an acceptable discriminatory accuracy for COVID-19 pneumonia in patients with BMI >30, while Adpn/Lep had an excellent discriminatory accuracy for COVID-19 pneumonia in patients with BMI <25, with a cutoff of 2.23. Our data indicate that high Adpn/Lep (>2.23) in lean patients is consistent with a state of good health, which decreases in case of inflammatory states, ranging from adipose tissue dysfunction with low-grade inflammation to COVID-19 pneumonia.

## 5. Conclusions

Our data reinforce the notion that high Adpn/Lep is an indicator of good metabolic health, as Adpn/Lep decreases in case of inflammatory states, ranging from the low-grade inflammation associated with metabolic syndrome to acute inflammatory states as in COVID-19 pneumonia.

## Figures and Tables

**Table 1:** Patient general characteristics.

Variables	Controls (*n* = 36)	COVID-19 patients (*n* = 48)	*p* value
Gender			
Male	23 (63.90%)	34 (70.80%)	0.50
Female	13 (36.10%)	14 (29.20%)	
Age (years)			
Median (min-max)	59.50 (36–82)	63.50 (34–88)	0.07
BMI (kg/m^2^)			
Median (min-max)	28.95 (19.14–45)	29.51 (20.76–41.03)	0.79
BMI <25	6 (16.67%)	10 (20.83%)	0.48
M/F	3/3 (50%; 50%)	6/4 (60%; 40%)	0.70
Median (min-max)	24 (19.14–24.69)	23.52 (20.76–24.80)	
25< BMI <30	14 (38.89%)	16 (33.33%)	0.31
M/F	11/3 (78.60%; 21.40%)	14/2 (87.50%; 12.50%)	0.51
Median (min-max)	27.34 (25–29.40)	27.78 (25.95–29.76)	
BMI >30	16 (44.44%)	22 (45.84%)	0.16
M/F	9/7 (56.20%; 43.80%)	14/8 (63.60%; 36.40%)	0.65
Median (min-max)	31.85 (30.50–45)	32.93 (30.12–41.03)	
Metabolic syndrome (yes)	16 (44.40%)	15 (31.20%)	0.22

BMI is for body mass index.

**Table 2:** Logistic multivariate regression models evaluating the association of leptin and Adpn/Lep with COVID-19 pneumonia.

	Dependent variable: presence of COVID-19 pneumonia		
Predictive variables	OR	95% CI	*p* value
Age	1.06	(1.01–1.12)	0.033
Sex (M)	3.18	(1.07–10.22)	0.043
BMI	0.93	(0.81–1.05)	0.257
Ln (leptin)	5.65	(2.00–19.81)	0.003
Age	1.05	(0.99–1.11)	0.087
Sex (M)	2.15	(0.76–6.42)	0.156
BMI	0.95	(0.83–1.09)	0.499
Adpn/Lep	0.51	(0.27–0.82)	0.019

Adpn/Lep is for adiponectin to leptin ratio; BMI is for body mass index; In is for natural logarithm; OR is for odds ratio; CI is for confidence interval.

**Table 3:** Linear association between adipokines and features of patients with COVID-19 pneumonia.

Variable	Leukocytes		Lymphocytes		Monocytes		NIMV (days)		Hospital (days)	
	Rho	*p*	Rho	*p*	Rho	*p*	Rho	*p*	Rho	*p*
Age	−0.02	0.86	−0.32	0.03	−0.13	0.38	0.15	0.31	0.49	<0.001
BMI	−0.28	0.05	0.03	0.86	−0.19	0.21	0.37	0.01	0.68	<0.001
CRP	0.16	0.26	−0.34	0.02	−0.09	0.54	0.21	0.16	0.12	0.40
Leptin	−0.07	0.64	0.24	0.10	0.10	0.48	0.25	0.08	0.13	0.39
Adiponectin	−0.16	0.26	0.10	0.51	−0.01	0.95	0.18	0.21	0.08	0.59
Adpn/Lep	−0.06	0.67	−0.15	0.30	−0.12	0.42	−0.14	0.34	−0.08	0.57
IL-6	−0.26	0.08	−0.32	0.03	−0.02	0.88	0.12	0.40	0.28	0.05
TNF-*α*	−0.30	0.04	0.03	0.82	−0.12	0.88	0.23	0.12	0.16	0.27

Adpn/Lep is for adiponectin to leptin ratio; BMI is for body mass index; CRP is for C-reactive protein; IL-6 is for interleukin-6; NIMV is for noninvasive mechanical ventilation; TNF-*α* is for tumor-necrosis factor-*α*. Spearman coefficient.

**Table 4:** Logistic multivariate regression models evaluating the association of BMI and leptin with maximum respiratory support.

	Dependent variable: use of VM/HFNC vs NIMV		
Predictive variables	OR	95% CI	*p* value
Age	1.10	(1.01–1.23)	0.048
Sex (M)	3.38	(0.51–31.85)	0.237
BMI	1.39	(1.09–1.96)	0.023
Leptin	1.00	(0.99–1.00)	0.491

BMI is for body mass index; NIMV is for noninvasive mechanical ventilation; VM/HFNC is for Venturi mask/high-flow nasal cannula (VM/HFNC); OR is for odds ratio; CI is for confidence interval.

## Data Availability

Some or all datasets generated and/or analyzed during the current study are not publicly available but are available from the corresponding author on reasonable request.
